# Lignin monomer composition affects *Arabidopsis *cell-wall degradability after liquid hot water pretreatment

**DOI:** 10.1186/1754-6834-3-27

**Published:** 2010-12-02

**Authors:** Xu Li, Eduardo Ximenes, Youngmi Kim, Mary Slininger, Richard Meilan, Michael Ladisch, Clint Chapple

**Affiliations:** 1 Department of Biochemistry, Purdue University, West Lafayette, IN 47907, USA; 2 Department of Agricultural and Biological Engineering and the Laboratory of Renewable Resources Engineering, Purdue University, West Lafayette, IN 47907, USA; 3 Department of Forestry and Natural Resources, Purdue University, West Lafayette, IN 47907, USA; 4 Weldon School of Biomedical Engineering, Purdue University, West Lafayette, IN 47907, USA

## Abstract

**Background:**

Lignin is embedded in the plant cell wall matrix, and impedes the enzymatic saccharification of lignocellulosic feedstocks. To investigate whether enzymatic digestibility of cell wall materials can be improved by altering the relative abundance of the two major lignin monomers, guaiacyl (G) and syringyl (S) subunits, we compared the degradability of cell wall material from wild-type *Arabidopsis thaliana *with a mutant line and a genetically modified line, the lignins of which are enriched in G and S subunits, respectively.

**Results:**

Arabidopsis tissue containing G- and S-rich lignins had the same saccharification performance as the wild type when subjected to enzyme hydrolysis without pretreatment. After a 24-hour incubation period, less than 30% of the total glucan was hydrolyzed. By contrast, when liquid hot water (LHW) pretreatment was included before enzyme hydrolysis, the S-lignin-rich tissue gave a much higher glucose yield than either the wild-type or G-lignin-rich tissue. Applying a hot-water washing step after the pretreatment did not lead to a further increase in final glucose yield, but the initial hydrolytic rate was doubled.

**Conclusions:**

Our analyses using the model plant *A. thaliana *revealed that lignin composition affects the enzymatic digestibility of LHW pretreated plant material. Pretreatment is more effective in enhancing the saccharification of *A. thaliana *cell walls that contain S-rich lignin. Increasing lignin S monomer content through genetic engineering may be a promising approach to increase the efficiency and reduce the cost of biomass to biofuel conversion.

## Background

Utilization of lignocellulosic biomass for biofuel production requires the hydrolysis of cellulose and other cell-wall polysaccharides to their component monosaccharides. This process is affected by many structural and compositional characteristics of the biomass, including the presence of lignin, a phenolic polymer composed of three major types of building blocks: *p*-hydroxyphenyl (H), guaiacyl (G) and syringyl (S) units. The association between lignin and the recalcitrance of biomass materials has long been recognized in forage feeding and tree pulping practices, and led to earlier lignin-engineering efforts aimed at improving feedstock performance in these processes [1]. For example, it has been shown that lignin content (the total amount of lignin in the tissue) greatly influences forage digestibility [2], and that lignin composition (the relative ratio of its component subunits) strongly affects the efficiency of the chemical pulping process. Specifically, transgenic poplar trees with higher S/G ratios show a greatly enhanced pulping efficiency [3]. A recent analysis of transgenic alfalfa revealed that high lignin content is correlated with the recalcitrance of cell-wall materials to enzymatic saccharification during biofuel production [4]. There was little variation in the S:G ratios of these alfalfa lines and as a result, the effects of lignin composition on the efficiency of biomass to biofuel conversion remain to be determined.

To investigate the effect of S-lignin composition on biomass degradability, we chose to analyze two *A. thaliana *lines in which the activity or expression of ferulate 5-hydroxylase (F5H), a key enzyme required for the synthesis of S-lignin monomer, is eliminated or enhanced, respectively. Both lines have been generated previously and characterized in detail [5-8]. The *fah1-2 *mutant is defective in F5H and does not deposit S lignin, whereas overexpression of *F5H *under the control of the cinnamate 4-hydroxylase (C4H) promoter in the C4H:F5H transgenic line results in lignin with an S-unit content in excess of 90% [6]. These lines represent two extremes in lignin composition and are each distinct from the wild type, which deposits a G/S copolymer with an S-subunit content of approximately 20 mol%. Despite their lignin difference, these two lines show similar growth to that of wild-type Arabidopsis.

In the biomass to biofuel conversion processes, pretreatment is generally used to increase the accessibility of cell-wall polysaccharides to enzymes. Pressure-cooking in liquid hot water (LHW) has been shown to be a cost-effective pretreatment to enhance the enzymatic digestibility of cellulose in a variety of feedstocks [9-12]. In this study, we assessed the cell-wall degradability of mutant and transgenic *A. thaliana *lines by enzyme hydrolysis without pretreatment, after LHW pretreatment, or after LHW pretreatment followed by hot water washing. Our results indicate that increasing the proportion of S subunits in *Arabidopsis *lignin decreases the recalcitrance of cell walls to enzymatic hydrolysis.

## Results

### Composition analysis

Consistent with previously published results [6], the lignins of *fah1-2 *and C4H:F5H plants are dominated by G and S subunits, respectively (Table 1). In contrast to the drastic differences in lignin composition, other cell-wall components of *fah1-2 *and C4H:F5H plants are relatively unchanged (Table 1). Whereas *fah1-2 *is similar to the wild type in glucan, xylan and acetyl content, C4H:F5H has slightly lower values for these components. Arabinan was not detected in any of the samples analyzed.

**Table 1:** Derivatization followed by reductive cleavage (DRFC) lignin and compositional analysis of Arabidopsis thaliana samples^1^

Plant sample	DFRC lignin (mol%)			Percentage by dry mass			
	
	**H**^**2**^	**G**^**3**^	**S**^**4**^	Glucan	Xylan	Arabinan	Acetyl
Wild type	6.0 (1.3)	76.2 (2.2)	17.8 (2.9)	22.7 (0.1)	12.3 (0.2)	0.0 (0.0)	4.2 (0.1)
*fah1-2*	4.8 (0.4)	95.2 (0.4)	0.0 (0.0)	23.1 (0.2)	13.6 (0.2)	0.0 (0.0)	3.8 (0.1)
C4H:F5H	4.1 (1.4)	5.2 (0.5)	90.7 (1.8)	18.0 (0.1)	11.0 (0.1)	0.0 (0.0)	3.4 (0.1)

^**1**^Mean values of three biological replications are represented, with standard errors shown in brackets.

^2^H = *p*-hydroxyphenyl.

^3^G = guaiacyl,

^4^S = syringyl,

### Enzyme hydrolysis of untreated samples

**Figure 1:**
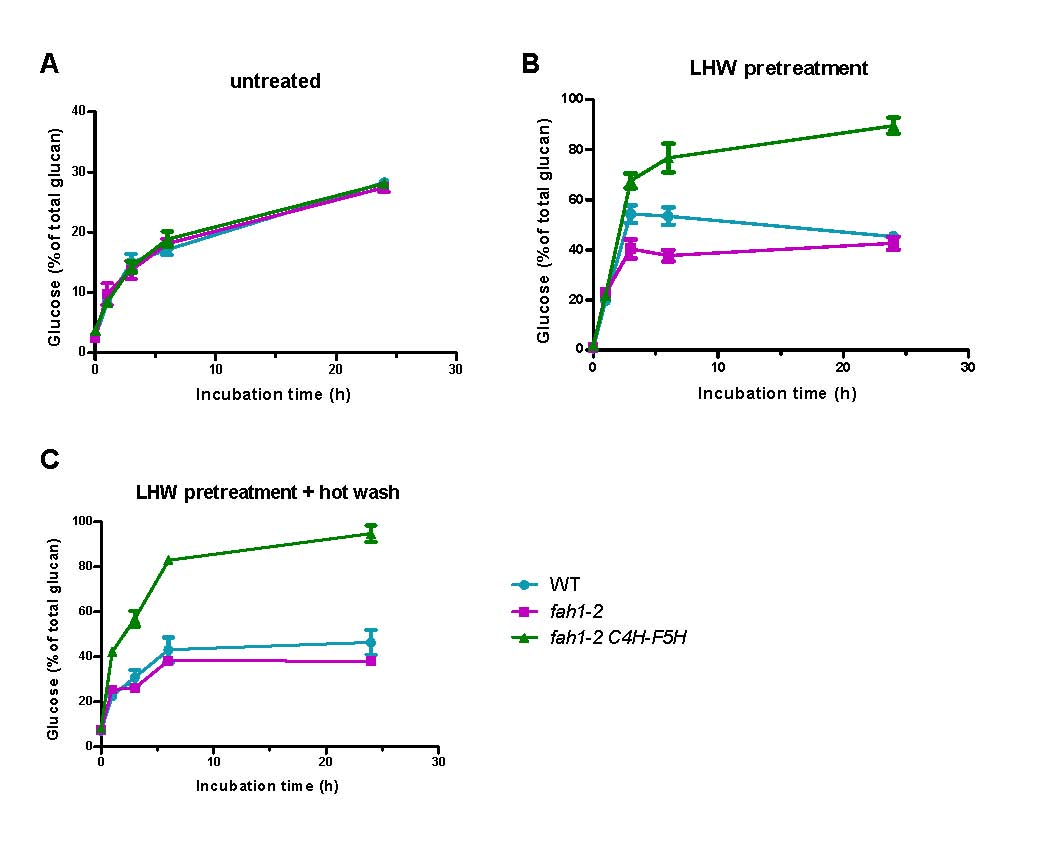
**Enzyme hydrolysis of stem material **. Enzyme hydrolysis time-course of **(A) **untreated, **(B) **liquid hot water (LHW)-pretreated, and **(B) **LHW-pretreated and hot-washed wild-type and genetically modified *Arabidopsis. thaliana *samples (2% (w/v) using a mixture of cellulase (Spezyme CP 50 filter paper units/g glucan or 90 mg protein/g glucan) and β-glucosidase (Novozyme 188 100 cellobiase units/g glucan or 34 mg protein/g glucan) at 50°C and pH 4.8.

As a first step to evaluate the possible effect of S lignin composition changes on cell-wall degradability, mature stems from wild-type, *fah1-2 *and C4H:F5H plants were subjected to enzyme hydrolysis without any pretreatment. There was limited cellulose hydrolysis in untreated stems (Figure 1A). After 24 hours of incubation, only about 30% of the maximum theoretical glucose yield was obtained for wild-type samples. The low- and high-S lignin samples have similar hydrolysis kinetics and glucose yield to those of the wild type, indicating that the structural changes to lignin in those plants do not have a significant effect on enzymatic hydrolysis of cellulose in untreated cell-wall materials.

### Enzyme hydrolysis with LHW pretreatment

We further tested the cell-wall degradability of the lines by applying LHW pretreatment before enzyme hydrolysis. Regardless of genotype, the hydrolytic rate and glucose yield of pretreated samples was greater than for the corresponding untreated samples (Figure 1B). Incubation of LHW-pretreated wild-type samples with enzymes for 1 hour released the same amount of glucose (20% of total glucan) as 6 hours of hydrolysis on untreated samples. More importantly, significant differences in cellulose hydrolysis were observed for LHW-pretreated *fah1-2 *and C4H:F5H samples (Figure 1B).No differences in glucose yield between the three genotypes were observed after 1 hour of incubation, but after 3 hours of incubation, C4H:F5H yielded more glucose and *fah1-2 *less glucose, compared with wild type. After 24 hours, the glucose yield of the C4H:F5H sample was nearly 90%.

### Scanning electronic microscopy

**Figure 2:**
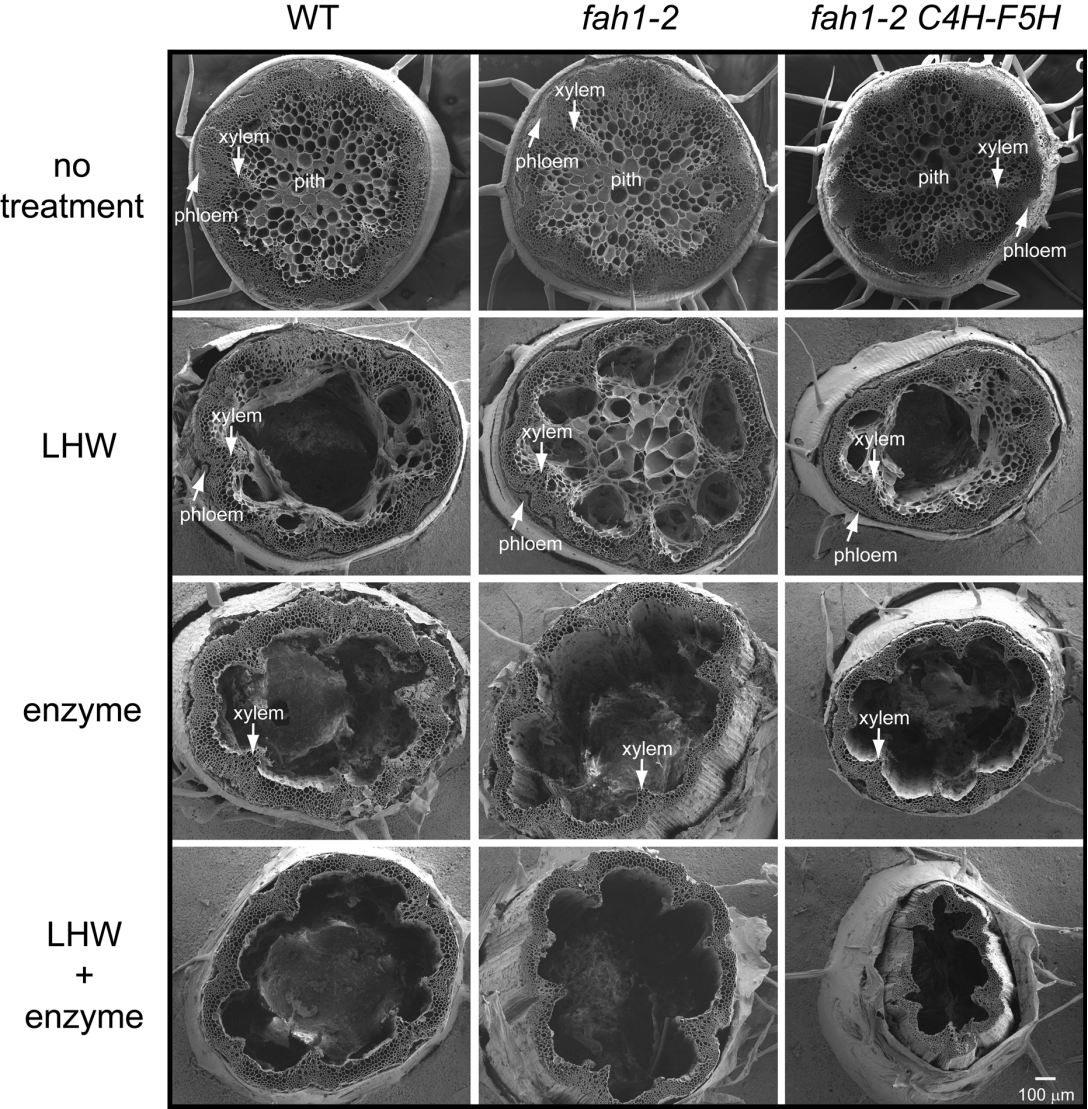
**Scanning electron microscopy (SEM) of stem cross-sections subjected to various treatments **. Wild-type, *fah1-2 *and C4H:F5H stems were cross-sectioned and affixed to a glass slide. Sections received either no treatment or one of the following treatments: liquid hot water (LHW) pretreatment, enzyme digestion, or LHW pretreatment plus enzyme digestion. Afterwards, they were imaged by SEM.

Pretreatment causes structural and/or chemical changes of biomass materials, making cellulose more accessible to hydrolytic enzymes [13]. To determine whether any anatomical changes occurred during LHW pretreatment and enzyme hydrolysis, we performed scanning electron microscopy (SEM) on stem cross-sections that had been subjected to different treatments. The wild-type, *fah1-2 *and C4H:F5H stems had similar vascular patterning (Figure 2). LHW pretreatment broke the pith cells and caused the detachment of the phloem from the xylem. This happened in all three genotypes, but *fah1-2 *had more intact pith cells compared with the wild type and C4H:F5H. In samples that underwent enzyme hydrolysis without pretreatment, both phloem and pith cells were completely disrupted, and there were no significant differences between the different genotypes. Interestingly, the stem cross-sections of C4H:F5H that had undergone LHW pretreatment plus enzyme hydrolysis were found to have collapsed, which was not observed in wild-type and *fah1-2 *samples after the same treatment.

Our enzyme hydrolysis results suggest that LHW pretreatment is differentially effective against *A. thaliana *samples with altered lignin composition.

### Enzyme hydrolysis after LHW pretreatment and hot-water washing

It has been shown that hot-water washing of LHW pretreated poplar can further improve its enzymatic digestibility [11]. Therefore, we also tested the cell-wall degradability of the *A. thaliana *samples after this additional step (Figure 1C). Unlike poplar, hot-water washing did not have a significant effect on the total glucose yield as calculated based on the initial glucan content of the Arabidopsis samples tested; however, the initial rate of cellulose hydrolysis of hot-water washed C4H:F5H samples was almost double that of unwashed samples. By contrast, hot-water washing has little effect on the initial hydrolytic rate of the wild-type and *fah1-2 *samples.

### Phenolic inhibitors analysis

The previous results suggest that hot-water washing may remove inhibitors of cellulase and/or β-glucosidase activity from C4H:F5H samples. To investigate this possibility, we analyzed the total content of phenolics in the liquid fraction after LHW pretreatment. All three genotypes had a similar concentration of total phenolics, approximately 0.3 mg/ml tannic acid equivalent (Table 2). This concentration is lower than the level known to significantly inhibit cellulose, although deactivation of these enzymes, particularly β-glucosidase may occur over a 24-hour period [14,15].

**Table 2:** Concentration of total phenolics in liquid collected after liquid hot water pre-treatment.^1,2^

*Arabidopsis *samples	**Concentration of total phenolics, mg TAE/ml**^**3**^
Wild type	0.30 ± 0.19
*fah1-2*	0.35 ± 0.01
C4H:F5H	0.30 ± 0.08

^**1**^Liquid was collected by centrifugation (3,000 rpm, 20 min) after liquid hot water pretreatment (200°C, 10 min and 30 sec) of Arabidopsis samples (2% (w/v)).

^**2**^Data are mean ± SE of three biological replications.

^**3**^TAE = tannic acid equivalent.

## Discussion

Lignin is a major contributor to the recalcitrance of biomass, and has been a target for feedstock improvement through genetic engineering. It has been demonstrated in several plant species that a reduction in lignin content using transgenic approaches enhances cell-wall degradability; however, significant improvement in conversion efficiency has often been accompanied by abnormal plant growth and development [1,4]. By contrast, plants seem to be amenable to wide ranges in lignin composition changes, including variation in the content of conventional monomers and the incorporation of atypical precursors [6,16-18]. Our findings of improved cell-wall degradability in *Arabidopsis *stems with high S-lignin content demonstrate the potential of lignin composition modification for the improvement of cellulosic feedstock performance.

LHW treatment had a dramatic effect on enzyme hydrolysis of biomass from the high S-lignin line. As much as 90% of the maximum theoretical glucose yield was achieved for C4H:F5H tissue, whereas less than 60% was obtained from wild-type material exposed to the same treatment. SEM analysis detected no obvious anatomical differences between the low and high S-lignin samples after LHW pretreatment. By contrast, after LHW pretreatment and enzyme hydrolysis, stem cross-sections from the high S-lignin stems had a distinct deformity, presumably due to enhanced hydrolysis of the cell wall.

How alteration of lignin composition relates to the observed increase in the effectiveness of LHW pretreatment is unclear. Recently, it was proposed that during high-temperature pretreatment, lignin is melted and relocalized to outer surface of the cell wall, increasing the accessibility of the cellulose within [19,20]. The S:G ratio is known to have profound effects on lignin structure. Whereas G-rich lignin has a branched structure, S-rich lignin is more linear and has a lower degree of polymerization [21]. It is tempting to speculate that S-rich lignin may have a lower melting point and is more easily relocated than G-rich lignin and, thereby, leads to improved enzymatic digestibility.

The observation that hot-water washing after LHW pretreatment significantly increases the initial saccharification rate of the high-S sample suggests removal of some inhibitory compounds. It has been shown LHW pretreatment of wet cake (solids left after corn is fermented to ethanol) releases some phenolics and water soluble xylo-oligosaccharides that can inhibit cellulases and β-glucosidases [14]. However, in this study, the concentration of phenolics in the liquid after LHW pretreatment was below the level reported to cause significant inhibition. Moreover, we did not see any difference in the concentration of phenolics between S-deficient, S-rich and wild-type *A. thaliana *tissue samples. Therefore, it is unlikely that the observed increase in saccharification rate was due to the removal of inhibitors. The relative influence of phenolic molecules on enzyme activity becomes more pronounced as the ratio of phenols to protein increases at higher solid and lower protein loadings than used in this study. Although protein loadings are currently in the range of 2 to 10 mg/g lignocellulose solids, which represent up to a five-fold decrease in enzyme from only 5 years ago, an even greater reduction is needed for economically viable processes [13,22].

It will be important to extend this study to other biomass feedstocks in the future. One of the factors that needs to be considered is the native lignin composition of different plant species, and possibly also the interaction of lignin with the polysaccharide components of the cell wall. It is likely that the dramatic increase in cell-wall degradability observed in *Arabidopsis *might be less apparent in plant species with high native S-lignin levels, such as hybrid poplar. However, significant increases in lignin extractability during the pulping process has been observed in S-lignin-enriched transgenic poplar [3], suggesting that the magnitude of S lignin increase may still contribute significantly to higher cell-wall degradability in this important biomass feedstock.

## Conclusions

The available genetically modified *A. thaliana *plants with different lignin composition and structure provided an opportunity to evaluate the possible effect of lignin modification on cell-wall recalcitrance. Our study revealed that high levels of S-lignin have a positive effect on the effectiveness of LHW pretreatment and enzymatic hydrolysis, at least in *Arabidopsis*. This effect might result from the physicochemical changes of lignin brought about by more linear structure of S subunits. In the future, it will be important to determine how widespread is this phenomenon and to elucidate its underlying mechanism.

## Methods

### Materials

Generation of the *A. thaliana fah1-2 *and C4H:F5H lines has been described previously [6,7]. The genetically modified and wild-type *A. thaliana *(Columbia) plants were grown side by side at 22°C under a 16-hour photoperiod. Mature stems were harvested by removing siliques and leaves.

Spezyme CP cellulase preparation from *Trichoderma reesei *containing exo-, endo- and β-glucosidase activities; batch number 3016295230) was provided by Genencor, Danisco Division (Palo Alto, CA, USA). Novozyme 188 (β-glucosidase from *Aspergillus niger*; catalogue number c6150) was purchased from Sigma Chemical Co. (St. Louis, MO, USA).

### Lignin analysis

Lignin composition was determined using the derivatization followed by reductive cleavage (DFRC) method [23].

### Compositional analysis

The composition of the plant samples was analyzed using standard National Renewable Energy Laboratory procedures [24]. To calculate polysaccharide composition, monomer sugars were analyzed by high-performance liquid chromatography (HPLC) after acid hydrolysis of the samples. HPLC analysis of liquid samples was performed on a system consisting of a solvent-delivery system (9010 Gradient HPLC Pump, Varian/Agilent, Santa Clara CA, USA), an autosampler (717 Plus; Waters Corp., Milford, MA, USA), a carbohydrate analysis column (Aminex HPX-87H; Bio-Rad, Hercules, CA, USA); a refractive index detector (2414; Waters Corp.) a dual-wavelength absorbance detector (2487; Waters Corp.); and an integrator (HP3396G; Hewlett Packard, Santa Clara CA, USA). The mobile phase was 5 mmol/l H_2_SO_4 _filtered through a 0.2 μm nylon filter (Millipore Corp., Billerica, MA, USA) and degassed. The mobile phase flow rate was 0.6 ml/min and the column temperature was maintained at 60°C by a column heater (CH-30; Eppendorf) with a temperature controller (TC-50; Eppendorf, Hauppauge, New York, USA).

### Cell-wall degradability analysis

The stem materials were ground for passage through a 20 mesh (841 μm) screen for cell-wall degradability analyses. Pretreatment was carried out by pressure cooking 50 mg samples in a metal tube containing 1.5 ml water at 200°C (30 seconds of heat-up time followed by a 10 minute hold). Each tube was placed in a fluidized sand bath (Tecam^® ^SBL-1; Cole-Parmer, Vernon Hills, IL). The pressure within the tubes was held at the saturation vapor pressure of water to keep the water in a liquid state [9,11-13]. The samples were cooled before the addition of 1.5 ml of 100 mmol/l citrate buffer pH 4.8, bringing the final volume to 3 ml (~2% solids (w/v)). For hot-water washing, samples were washed twice with 3 ml of 70°C water. After the second wash, no glucose was detected. The enzyme hydrolysis for all the conditions tested was based on initial solids loading and glucan concentration. Commercial cellulase (Spezyme CP) at 0.2 filter paper units (FPU)/ml or 50 FPU/g glucan (90 mg protein/g glucan) and β-glucosidase (Novozyme 188) at 0.35 cellobiase units (CBU)/ml or 105 CBU/g glucan (34 mg protein/g glucan) were added, and hydrolysis was carried out for different lengths of time at 50°C and pH 4.8 in an incubator shaker (New Brunswick Scientific, Edison, NJ, USA). The ratio of enzyme to solids was equivalent to 10 FPU/g total solids, 21 CBU/g total solids and 25 mg protein/g total solids. Enzyme hydrolysis of 50 mg untreated samples (also at ~2% solids (w/v), 50°C and pH 4.8) was carried out under similar experimental conditions.

### SEM analysis

Stem cross-sections were adhered to a glass slide with epoxy adhesive (J-B Weld; J-B Weld Co., Sulphur Springs, TX, USA) and subjected to different treatments. Pretreatment was performed as described in the previous section. When applied, enzyme hydrolysis was carried out with a two-fold increase in enzyme loading to compensate for the particle size differences between the samples used for SEM and cell-wall degradability analysis. Subsequently, the stem cross-sections were fixed in two steps: first with a mixture of 2% paraformaldehyde and 2.5% glutaraldehyde in 0.1 mol/l cacodylate buffer, pH 7.4 for 1 hour and then with 1% OsO_4 _in 0.1 mol/l cacodylate buffer, pH 7.4 for 30 minutes. After critical point drying, the samples were sputter-coated with gold, and viewed under SEM (Nova nanoSEM; Fei Co., Hillsboro, OR, USA).

### Protein and phenolic measurements

The protein content of the commercial enzyme preparations was determined using a commercial kit (Pierce BCA Protein Assay Kit; product number 23225; Thermo Scientific, Rockford, IL, USA). Phenolic compounds were assayed using Prussian blue [25]. The diluted sample liquid (3 ml aliquots) was transferred to a 10 mm cuvette, then 200 μL of 0.008 mol/l K_2_Fe(CN)_6 _were added, followed by the immediate addition of 200 μL of 0.1 mol/l FeCl_3 _in 0.1 mol/l HCl. Absorbance was read at 700 nm after 5 min at room temperature, against a tannic acid standard.

## Competing interests

Michael Ladisch is Chief Technology Officer at Mascoma Corporation. Clint Chapple is the inventor on patents related to engineering S lignin content in plants.

## Authors' contributions

XL carried out the lignin analysis, participated in SEM imaging, and drafted the manuscript. EX participated in SEM imaging and helped to draft the manuscript. EX and MS carried out cell-wall degradability analyses. YK and MS carried out cell-wall compositional analysis and phenolic measurements. XL and CC conceived the study. CC, RM and ML participated in its design and coordination, and helped to draft the manuscript. All authors read and approved the final manuscript.
